# Evaluating the Impact of Stopping Chronic Therapies after Modulator Drug Therapy in Cystic Fibrosis: The SIMPLIFY Clinical Trial Study Design

**DOI:** 10.1513/AnnalsATS.202010-1336SD

**Published:** 2021-03-30

**Authors:** Nicole Mayer-Hamblett, David P. Nichols, Katherine Odem-Davis, Kristin A. Riekert, Greg S. Sawicki, Scott H. Donaldson, Felix Ratjen, Michael W. Konstan, Noah Simon, Daniel B. Rosenbluth, George Retsch-Bogart, John P. Clancy, Jill M. VanDalfsen, Rachael Buckingham, Alex H. Gifford

**Affiliations:** ^1^ Cystic Fibrosis Therapeutics Development Network Coordinating Center, Seattle Children’s Hospital, Seattle, Washington;; ^2^ Department of Pediatrics and; ^3^ Department of Biostatistics, University of Washington, Seattle, Washington;; ^4^ Division of Pulmonary and Critical Care Medicine, Department of Medicine, School of Medicine, Johns Hopkins University, Baltimore, Maryland;; ^5^ Division of General Pediatrics and Respiratory Diseases, Boston Children’s Hospital, Boston, Massachusetts;; ^6^ Department of Pediatrics, Harvard Medical School, Harvard University, Boston, Massachusetts;; ^7^ Division of Pulmonary and Critical Care Medicine and; ^12^ Division of Pediatric Pulmonology, University of North Carolina, Chapel Hill, Chapel Hill, North Carolina;; ^8^ Division of Respiratory Translational Medicine Program, SickKids Research Institute, Medicine, The Hospital for Sick Children, Toronto, Ontario, Canada;; ^9^ Department of Paediatrics, University of Toronto, Toronto, Ontario, Canada;; ^10^ Department of Pediatrics, Rainbow Babies and Children’s Hospital and School of Medicine, Case Western Reserve University, Cleveland, Ohio;; ^11^ Division of Pulmonary and Critical Care Medicine, Department of Medicine, School of Medicine, Washington University, St. Louis, Missouri;; ^13^ Department of Pediatrics, Cincinnati Children’s Hospital Medical Center, Cincinnati, Ohio;; ^14^ Cystic Fibrosis Foundation, Bethesda, Maryland;; ^15^ Pulmonary and Critical Care Medicine, Dartmouth-Hitchcock Medical Center, Lebanon, New Hampshire; and; ^16^ The Dartmouth Institute for Health Policy and Clinical Practice, Lebanon, New Hampshire

**Keywords:** CFTR modulators, treatment burden, noninferiority trial

## Abstract

The care for individuals with cystic fibrosis (CF) with at least one F508del mutation will greatly change as a result of the unparalleled clinical benefits observed with the new triple-combination CFTR (CF transmembrane regulator)–modulator therapy elexacaftor/tezacaftor/ivacaftor (ETI). Incorporating ETI into the standard of care creates new motivation and opportunity to consider reductions in overall treatment burden and evaluate whether other chronic medications can now be safely discontinued without loss of clinical benefit. SIMPLIFY is a master protocol poised to test the impact of discontinuing versus continuing two commonly used chronic therapies in people with CF who are at least 12 years of age or older and stable on ETI therapy. The protocol is composed of two concurrent randomized controlled trials designed to evaluate the independent short-term effects of discontinuing hypertonic saline or dornase alfa, enabling individuals on both therapies to participate in one or both trials. The primary objective for each trial is to determine whether discontinuing treatment is *noninferior* to continuing treatment after establishment of ETI, as measured by the 6-week absolute change in the percent-predicted forced expiratory volume in 1 second. Developing this study required a balance between ideal study-design principles and feasibility. SIMPLIFY will be the largest multicenter, randomized, controlled medication-withdrawal study in CF. This study is uniquely positioned to provide timely evidence on whether the daily treatment burden can be reduced among individuals on CFTR-modulator therapy.

Clinical trial registered with www.clinicaltrials.gov (NCT 04378153).

The landscape of clinical care and treatment for individuals with cystic fibrosis (CF) is poised for remarkable change after the discovery of a novel disease-modifying therapy that restores protein function to the CFTR (CF transmembrane regulator) (1). Elexacaftor/tezacaftor/ivacaftor (ETI) is a CFTR-modulator combination that has demonstrated clinical benefits for individuals with CF harboring at least one copy of the common F508del mutation. Assuming widespread approval and access, ETI therapy is likely to become a standard treatment option for approximately 90% of the CF population. Phase 3 clinical trials of ETI established substantial improvements in pulmonary function, reductions in pulmonary exacerbations and patient-reported respiratory symptoms, and improvements in nutritional status among individuals treated with ETI as compared with placebo (2, 3). ETI is expected to have sustained long-term clinical benefits similar to those of ivacaftor (4–6), a drug that has been approved for approximately 8 years for individuals with CF with less common mutations representing <10% of the CF population.

A high treatment burden and complexity have led people with CF and their providers to call for research identifying ways to reduce the overall treatment burden without sacrificing the incremental health gains achieved through the addition of therapies over time (7–9). The impactful clinical benefits of ETI provide new motivation to address the pressing question of whether certain chronic therapies targeted at managing symptoms and sequelae of the disease can now be withdrawn without clinical consequence after establishment of ETI therapy. Embarking on a new roadmap of CF clinical research evaluating withdrawing, rather than adding, chronic therapies cannot not be achieved without the collective engagement and input from the broader CF community. To inform this research, community stakeholders from the CF Foundation (CFF) Community Voice group were engaged in a focus group including two adults with CF and a parent of a child with CF (10). Feedback from this group was used to develop a comprehensive survey for the CF community and their providers collecting input on the necessity for a randomized controlled trial, preferred therapies to consider for withdrawal, and other key study-design details such as study duration and meaningful trial outcomes. This survey elicited overwhelming support for a randomized trial of treatment withdrawal in the context of highly effective modulator therapy (10).

A rigorous evaluation of the impact of treatment withdrawal through a randomized controlled trial is an ideal approach to assess both safety and efficacy outcomes from a framework of clinical equipoise and mitigates the risks of confounding through indication bias inherent in observational study designs. There are limited examples of treatment-withdrawal trials for chronic therapies in either CF or other diseases. To date, the most notable example in CF was a multicenter, randomized withdrawal trial of inhaled corticosteroids (11). Although the trial demonstrated no safety concerns associated with withdrawal of corticosteroids, it did not provide sufficient power to definitively state that there was no significant impact on the risk of pulmonary exacerbation associated with withdrawal. This prior study underscores the importance of adequately powered trials testing for noninferiority if one is concerned both with the risk of even relatively small declines in health after withdrawing a chronic therapy and the desire to state that two treatment regimens are comparable if no such declines exist. Withdrawal studies will inherently require large numbers of participants and the need to consider feasibility in the setting of a rare disease such as CF.

To address the need for timely and rigorous evaluation of chronic medication withdrawal in CF in the context of ETI use and fewer daily symptoms of disease, the SIMPLIFY protocol was developed: it is a master protocol including two randomized controlled trials to test the impact of discontinuing versus continuing chronic therapies in people with CF on highly effective CFTR-modulator therapy. SIMPLIFY investigates the withdrawal of two relatively burdensome and commonly used inhaled medications (10), hypertonic saline and dornase alfa, which have demonstrated short-term effects on measures of pulmonary function among those not treated with modulator drugs (12–16). Although airway-clearance therapy and inhaled antibiotics were ranked as slightly more burdensome than hypertonic saline and dornase alfa among 667 CF community members completing our initial survey (10), the heterogeneity of use and greater uncertainty in selecting informative clinical outcome measures for both airway-clearance therapies and inhaled antibiotics add significant complexity to withdrawal trial design. A total of 218 clinicians responding to the same survey ranked hypertonic saline and dornase alfa as the top two therapies to include in a withdrawal study over airway-clearance therapy, inhaled antibiotics, and macrolides. These two mucolytic therapies are favorable candidates for a withdrawal trial design, given their widespread chronic use, overlapping physiological effects with modulator drugs, and relatively high treatment burden. Furthermore, it is currently unknown whether hypertonic saline or dornase alfa will improve or maintain pulmonary health above what is gained through ETI.

SIMPLIFY was designed to independently test the effect of discontinuing hypertonic saline or dornase alfa as compared with continuing each therapy, hypothesizing no clinically meaningful short-term impact on lung function or safety outcomes would occur between those discontinuing therapy and those continuing therapy. The design of the SIMPLIFY trial required complex consideration to balance ideal design principles with feasibility. Here we report the SIMPLIFY design and the key considerations informing the largest medication-withdrawal study in CF. Further study details, including the Standard Protocol Items: Recommendations for Interventional Trials checklist and a schedule of assessments, are provided in the online supplement (17).

## Study Sites and Coordination

The SIMPLIFY study is sponsored by the CFF and is currently enrolling across 80 participating adult and pediatric centers in the CF Therapeutics Development Network (TDN). The TDN consists of 91 research centers across the United States and a coordinating center in Seattle, Washington, dedicated to conducting studies to cure and control CF (18). A data-monitoring committee independent under the CF Data Safety Monitoring Board provides ongoing safety monitoring.

## Study-Design Overview

**Figure 1.**
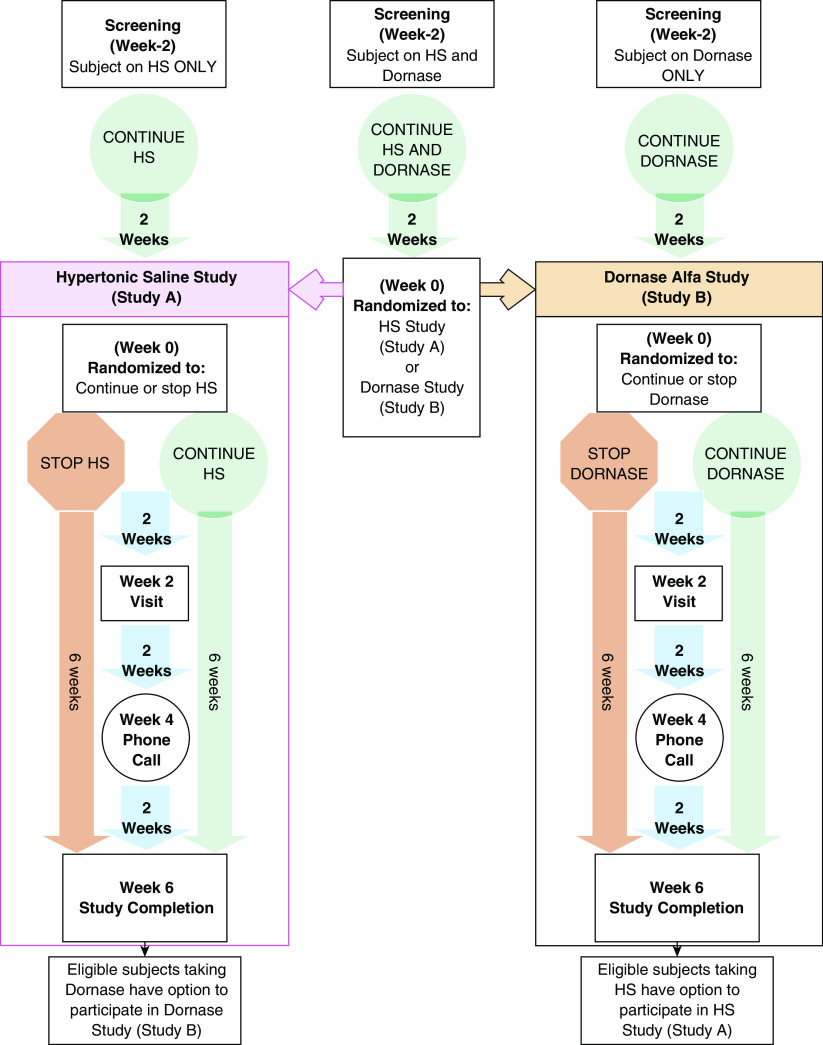
SIMPLIFY study design schematic. Study A and study B are identical randomized, open-label, two-arm trials consisting of a 2-week screening period and randomization to either continue or discontinue hypertonic saline (HS) (study A) or dornase alfa (Dornase) (study B), followed by a 6-week study period. Study visits occur at Weeks −2 (screening), 0, 2, and 6. Only those who maintain adequate reported adherence to inhaled drug therapy between screening (Week −2) and Week 0 are eligible for randomization (Table 1). At Week 0, subjects currently being treated with only HS or Dornase will be enrolled in study A or study B (as applicable) and will be randomized 1:1 to either continue or discontinue their current prescribed therapy. At study entry, subjects who are currently being treated with both HS and Dornase will remain on both therapies during the screening period and then be randomized to study A (HS) or study B (Dornase) as well as being randomized (1:1) to continue versus discontinue the applicable therapy. The randomization to study A or study B among subjects on both therapies is not optional and is essential to reduce indication bias and ensure comparable populations across studies. After completion of the first study, these subjects may subsequently enroll in the alternative study if they meet eligibility criteria. Reenrolling subjects need not remain on the treatment regimen assigned in the first study but must meet all eligibility criteria regarding treatment stability before entry (Table 1). Within each study, randomization will be stratified by the Week 0 percent-predicted forced expiratory volume in 1 second (⩾90, <90), treatment combination at screening (single or concurrent use of HS and/or Dornase), prior study participation (yes/no), and age (⩾18 versus <18). For subjects randomly assigned to continue their therapy during a given study, this therapy is expected to be taken at least once daily according to each subject’s preexisting, clinically prescribed regimen. If one study completes enrollment faster than the other study, the protocol will be restricted to enrollment in only the open study.

SIMPLIFY is a master protocol with two concurrent, 6-week, randomized controlled trials (Figure 1), each designed to evaluate the independent effect of discontinuing hypertonic saline (study A) or dornase alfa (study B). Individuals with CF of ages 12–17 years with a percent-predicted forced expiratory volume in 1 second (ppFEV_1_) ⩾ 70 and those 18 years and older with a ppFEV_1_ ⩾ 60 may enroll if they have been on ETI and either or both mucolytic therapies (⩾3% hypertonic saline and/or dornase alfa) for at least 90 days before screening. Study A will enroll approximately 400 subjects (approximately 200 randomized to discontinue hypertonic saline and 200 randomized to continue hypertonic saline). Study B will enroll approximately 400 subjects (approximately 200 randomized to discontinue dornase alfa and 200 randomized to continue dornase alfa). Subjects on both hypertonic saline and dornase alfa may participate sequentially in both studies. Additional eligibility criteria for the study population are listed in Table 1, and study-design details are provided in both Figure 1 and the online supplement.

**Table 1.** Eligibility criteria for SIMPLIFY

Eligibility criteria at screening (Week −2). Eligibility criteria will be evaluated at the screening visit (Week −2) for each study in the protocol. Subjects who enter the SIMPLIFY master protocol receiving only hypertonic saline or only dornase alfa at the time of entry will only be eligible to participate in one study.
*Consent*
• Written informed consent (and assent when applicable) obtained from subject or subject's legal guardian
• Enrolled in the CF Patient Registry
• For the 6-wk study duration, willingness to either continue or discontinue daily use of hypertonic saline or dornase alfa (as applicable to study A or study B) based on randomization and according to the clinically prescribed routine (i.e., at least once daily)
• Is willing and able to adhere to the study-visit schedule and other protocol requirements, including willingness and ability to provide information using electronic questionnaires loaded onto a personal device (e.g., smartphone or tablet)
• For subjects who enter the SIMPLIFY master protocol receiving both hypertonic saline and dornase alfa at the time of entry into their first study: willingness to be randomized to either study A or study B
*Demographics*
• Age ⩾ 12 yr at the screening visit
*Disease history*
• Diagnosis of CF
• ppFEV_1_ ⩾ 70 at the screening visit if <18 yr old and ppFEV_1_ ⩾ 60 at screening visit if ⩾18 yr old
• After interim analysis, if the DMC approves, a separate cohort (lower-lung-function cohort) of approximately 120 subjects ⩾18 yr old with a ppFEV_1_ of 40 to <60 will be enrolled into study A.
• Clinically stable with no significant changes in health status within the 7 d prior to and including the screening visit
• No active smoking or vaping
• Has no other conditions that, in the opinion of the site investigator/designee, would preclude informed consent or assent, make study participation unsafe, complicate interpretation of study-outcome data, or otherwise interfere with achieving the study objectives
*Concomitant medications and treatments*
• Current treatment with ETI for at least the 90 d before and including the screening visit and willing to continue daily use for the duration of the study
• Currently receiving hypertonic saline (at least 3%) and/or dornase alfa for at least the 90 d before and including the screening visit and willing to continue daily use for the 2-wk screening period*
• Ability to tolerate albuterol or levalbuterol (Xopenex)
• No use of an investigational drug within the 28 d before and including the screening visit
• No changes to chronic therapy (e.g., ibuprofen, azithromycin, inhaled tobramycin, aztreonam lysine) within the 28 d before and including the screening visit. This includes new airway-clearance routines
• No acute use of antibiotics (oral, inhaled, or IV) or acute use of systemic corticosteroids for respiratory-tract symptoms within the 7 d before and including the screening visit
• No chronic use of systemic corticosteroids at a dose equivalent to ⩾10 mg/d of prednisone within the 28 d before and including the screening visit
• No antibiotic treatment for NTM within the 28 d before and including the screening visit

Eligibility criteria at randomization (Week 0). Eligibility criteria will be evaluated before randomization at visit 1 (Week 0) for each study.
*Consent*
• Is willing and able to adhere to the study-visit schedule and other protocol requirements
*Disease history*
• No absolute decrease in ppFEV_1_ ⩾10 between the screening visit and visit 1 (Week 0)
• Clinically stable with no significant changes in health status between the screening visit and visit 1 (Week 0)
*Concomitant medications and treatments*
• No acute use of antibiotics (oral, inhaled, or IV) or acute use of systemic corticosteroids for respiratory-tract symptoms from the screening visit to visit 1 (Week 0)
• More than 70% compliance with submission of daily ePRO questionnaires in the up to 13 d before visit 1 (Week 0)
• Among the daily ePRO questionnaires submitted in the up to 13 d before visit 1, at least 70% adherence with receiving ETI and, as applicable, hypertonic saline and/or dornase alfa, as reported from screening to visit 1 (Week 0)

Additional eligibility for MCC substudy
• Able to perform the testing and procedures required for the study, as judged by the investigator
• Able and willing to withhold hypertonic saline and dornase alfa for at least 12 h before each MCC scan at visits 1 (Week 0) and 3 (Week 6)
• Those able to become pregnant: negative pregnancy test at visit 1 (Week 0)
• Those able to become pregnant: able and willing to practice a medically acceptable form of contraception from 3 d before visit 1 (Week 0) through visit 3 (Week 6) (acceptable forms of contraception: hormonal birth control, intrauterine device, barrier method plus a spermicidal agent, or abstinence) unless surgically sterilized or postmenopausal
• No more than two chest CT scans in the 12 mo before visit 1 (Week 0) (or a combination of procedures that are believed to have exposed the subject’s lungs to >150 mSv for adults ⩾18 yr old or >15 mSv for children <18 yr old)

*Definition of abbreviations*: CF = cystic fibrosis; CT = computed tomography; DMC = data-monitoring committee; ePRO = electronic patient-reported outcome; ETI = elexacaftor/tezacaftor/ivacaftor; IV = intravenous; MCC = mucociliary clearance; NTM = nontuberculous mycobacteria; ppFEV_1_ = percent-predicted forced expiratory volume in 1 second.

These eligibility criteria must be met for all participants, regardless of prior study participation.

* For participants with prior participation in SIMPLIFY, subjects must continue with assigned use/nonuse of therapy in the prior trial or reestablish consistent hypertonic saline and/or dornase-alfa therapy before entering into the second study. There are no time constraints for reentering.

For each study (A and B), clinical and safety outcomes, including ppFEV_1_, antibiotic use, pulmonary exacerbations, adverse events, and patient-reported outcomes, will be evaluated at all sites at each study visit and/or over the duration of the study for event-based outcomes. Additional measurements will be conducted at selected sites with the capabilities to conduct these procedures: multiple-breath washout at Weeks −2, 0, and 6 among approximately 400 subjects across both studies to evaluate changes in the lung-clearance index (LCI) and mucociliary-clearance (MCC) scans among approximately 60 subjects across both studies using inhaled radiolabeled particles and imaging techniques at Weeks 0 and 6 to evaluate changes in MCC. All subjects will in addition be asked to complete electronic participation questionnaires at the completion of each study regarding the use of ETI, hypertonic saline, dornase alfa, mechanical airway-clearance therapy, and antibiotics approximately every 28 days for up to 24 weeks after their final study visit at Week 6. For subjects who participate in a second subsequent trial within SIMPLIFY within 24 weeks of completing the first, the post-study questionnaires from the first study will be stopped early.

An optional cohort of 120 additional ETI-treated subjects ⩾18 years old with lower baseline lung function (ppFEV_1_ of 40–59) may be enrolled into study A (hypertonic saline) upon interim review of safety data by the data-monitoring committee after at least 25% enrollment is complete (Table 1). The decision to expand enrollment into study A only was based on prioritization of study and patient resources. Outcomes will be independently evaluated in this cohort with a focus on safety.

## Blinding and Adherence to Treatment Assignment

Blinding was a major factor for consideration in the study design and, although desirable, was determined not to be feasible because of the inability to acquire a suitable placebo for both hypertonic saline and dornase alfa. Prior placebo-controlled trials of hypertonic saline relied on lower concentrations of saline to serve as a placebo, yet this is not equivalent to stopping therapy completely. In addition, lowering concentrations of the saline for individuals already accustomed to using this medication would likely be noticeable, given the taste and airway sensation induced by higher but not lower salt concentrations. Dornase alfa is a biological, commercially approved compound with complex drug-production requirements. Obtaining a matched placebo with a similar aerosolized taste and appearance would significantly increase trial cost, complexity, and time to begin enrolling. It was decided that the benefits of placebo-controlled blinding did not outweigh these considerations in a study with several objective outcome measures, including the primary outcome of ppFEV_1_.

Given the practical limitations to blinding the trial, designing mechanisms to promote, monitor, and document adherence to the treatment assignment, including limiting each study to a 6-week duration, was a key focus in the design phases. The approach for monitoring, however, needed to be implemented in a consistent fashion across both treatment groups to be unbiased and not induce changes in therapeutic dosage or patterns of adherence simply due to the mechanism of data capture itself. For this reason, adherence methods that could only be performed in the treatment-continuation arm (e.g., nebulizers recording use data) were not incorporated. Rather, with input from the CFF’s Success with Therapies Research Consortium, SIMPLIFY was designed to use time-triggered ecological momentary assessment through the deployment of standardized daily electronic questionnaires to all participants, regardless of treatment assignment (19–22). These daily assessments document use of ETI, hypertonic saline, dornase alfa, and mechanical airway-clearance therapy for all subjects, regardless of whether they are randomized to continue or stop a therapy. Participants must also demonstrate adequate treatment adherence and data reporting using the electronic questionnaires during a 2-week run-in period to be eligible for randomization. After randomization, the data will be used for assessing adherence to the randomized treatment assignment and derivation of analysis populations as described further below. Although there is no masking to treatment assignment for individual subjects or their clinicians, aggregate study results are blinded and tightly controlled by the data-coordinating center.

## Objectives and Endpoints

The primary objective is evaluated for each study separately and is to determine whether discontinuing treatment is *noninferior* to continuing treatment after establishment of chronic ETI, as measured by the 6-week mean absolute change in ppFEV_1_. The choice of primary endpoint was informed by clinicians who ranked decreases in lung function as the most important indicator of health deterioration in people with CF (10), and a 6-week time point was chosen to support a practical, feasible study-visit schedule promoting adherence with the treatment regimen and enabling sufficient washout (12). Secondary objectives include evaluating the safety of discontinuing compared with continuing treatment as measured through adverse events and evaluating the effects on the change in the LCI and clinical outcomes, including the frequency of acute antibiotic usage, pulmonary exacerbations, and patient-reported outcome scores. The effect on patient perception of total inhaled treatment time will also be evaluated in addition to the exploratory endpoint evaluating the 6-week change in MCC. A complete list of outcomes for each study is provided in Table 2.

**Table 2.** Overview of SIMPLIFY study endpoints

Primary endpoint
The primary endpoint in each study is the mean absolute change in ppFEV_1_ from visit 1 (Week 0) to visit 3 (Week 6).
Secondary endpoints
Efficacy
• Mean change in LCI from visit 1 (Week 0) to visit 3 (Week 6)
• Mean change in ppFEV_1_ from visit 1 (Week 0) to visit 2 (Week 2)
• Proportion of subjects initiating acute antibiotics from visit 1 (Week 0) to visit 3 (Week 6)
• Proportion of subjects hospitalized from visit 1 (Week 0) to visit 3 (Week 6)
• Proportion of subjects with a pulmonary exacerbation from visit 1 (Week 0) to visit 3 (Week 6), defined according to expanded Fuchs criteria (31)
• Mean change in CRISS (32) from visit 1 (Week 0) to visit 3 (Week 6)
• Mean change in the CFQR respiratory-domain score (33) from visit 1 (Week 0) to visit 3 (Week 6)
• Mean change in ppFEV_1_ from screening to visit 1 (Week 0)
Safety
• Incidence of adverse events occurring between visit 1 (Week 0) to visit 3 (Week 6)
• Proportion of subjects temporarily or permanently changing their assigned therapy regimen between visit 1 (Week 0) to visit 3 (Week 6)
Exploratory endpoints
• Mean change in MCC from visit 1 (Week 0) to visit 3 (Week 6)
• Proportions of subjects remaining on and off hypertonic saline and dornase alfa for up to 24 wk after completion of each study
• Proportions of subjects with acute antibiotic use up to 24 wk after completion of each study
• Average impact score at visit 3 (Week 6) on subject perception of how stopping hypertonic saline or dornase alfa (or both) would impact their daily life

*Definition of abbreviations*: CFQR = Cystic Fibrosis Questionnaire–Revised; CRISS = Chronic Respiratory Infection Symptom Score; LCI = lung-clearance index; MCC = mucociliary clearance; ppFEV_1_ = percent-predicted forced expiratory volume in 1 second.

Additional exploratory objectives include estimating and comparing the effects of discontinuing versus continuing both hypertonic saline and dornase alfa among the subgroup of subjects using both chronic therapies and evaluating the association between the decision to remain on or off therapy up to 24 weeks after the study with both randomization assignment and study outcomes. A unique feature of the SIMPLIFY design is the potential to pool data across the identically designed individual trials to address several of these ancillary research questions.

## Primary Statistical Plan and Sample-Size Considerations

The primary hypothesis for both study A and study B is that discontinuing therapy is noninferior to continuing therapy, as measured by the 6-week change in the ppFEV_1_. The primary analysis will be conducted on the per-protocol population defined by criteria including ⩾70% adherence to the assigned treatment regimen after randomization and no major *a priori* defined protocol deviations and will be repeated as a sensitivity analysis in the intent-to-treat population. For each study, a linear regression model will be used to adjust for randomization strata and generate estimated effects of discontinuation versus continuation of therapy with corresponding 95% confidence intervals. An *a priori* noninferiority margin of −3 ppFEV_1_ was established for each study on the basis of clinical consensus during the scientific review of the protocol. Noninferiority will be claimed for each study if the lower bound of the 95% confidence interval for the difference between treatment arms in the 6-week absolute change in ppFEV_1_ is greater than −3, ruling out clinically significant acute changes in lung function.

Data from previous studies were used to estimate the standard deviation of change in ppFEV_1_, resulting in an estimate of 8.4 per group (2, 3). It is anticipated from prior CF trials conducted through the TDN that the attrition and nonadherence rate will be less than 20%, and it is thus reasonable to expect that a total sample size of 400 per study will enable at least 308 subjects to complete the trial and be included in the per-protocol population. A total sample size of 308 provides 88% power to detect noninferiority with a margin of −3 ppFEV_1_ when there is truly no effect of discontinuation. It provides 74% power when, in truth, discontinuation results in an average absolute decrease in the ppFEV_1_ of 0.5. Assuming a standard deviation of 8.4 and per-protocol sample size of 154 per group, the largest observed decrease in ppFEV_1_ comparing the discontinuation arm to the continuation arm that would meet noninferiority criteria would be approximately −1.12.

## Trial Status

The trial protocol was approved by the institutional review board in March of 2020, and the first site was activated for enrollment in August of 2020. The TDN extensively tracked the status of clinical research across the network and initiated the start of the trial when over 90% of sites reported reopening for new interventional studies. No adaptations were made to the trial protocol for coronavirus disease (COVID-19), and enrollment was thus limited to sites able to collect the key study endpoints via in-person clinic visits, with particular attention paid to abiding by institutional guidelines for aerosol-generating procedures. Although clinical research has shifted toward telemedicine during the pandemic and adoption of remote data collection for endpoints such as pulmonary function, prior data from CF trials indicate that there may be significantly increased variability in the measurement of the average change in lung function using handheld spirometers at home as compared with using clinic spirometry. This increased variability alone, independent of any potential systemic bias induced by using remote versus clinic-based spirometry, would have a significant impact on the planned sample size for the trial (23). Initial enrollment success observed with SIMPLIFY (*see* Figure E1 in the online supplement) demonstrates the ability of the sites to overcome COVID-19 restrictions and the enthusiasm of the CF community to address the research questions posed in SIMPLIFY.

## Discussion

SIMPLIFY is the largest medication-withdrawal study in CF to date, motivated by the increasing availability of effective CFTR-modulator therapy to a majority of the CF population, and is poised to test the hypothesis that there is no meaningful impact on short-term clinical outcomes or safety associated with discontinuation of hypertonic saline or dornase alfa. The 6-week duration was established to promote feasibility and treatment-regimen adherence during the study. Notably, repeated 14-day cycles of dornase alfa use and washout demonstrated dynamic improvement and a return to the baseline ppFEV_1_ within this timeframe. Although the ppFEV_1_ has been the most frequently used efficacy outcome measure of lung function in CF clinical trials (24), an alternative and perhaps more sensitive measure of change in lung function using the multiple-breath washout LCI has been used to test short-term pulmonary effects of mucoactive drugs or modulators (15, 16, 25). The potential to capture even small changes in pulmonary function through the LCI, perhaps more significantly among those with higher baseline lung function, will increase the ability of SIMPLIFY to detect and understand the impact of treatment withdrawal on airway function. In addition, MCC scans from a subset of participants will provide important complementary physiological data that are relevant to the mechanism of these medications (13, 26–28). Consistency across all three of these related outcome measures would increase confidence in the interpretation of the trial results.

SIMPLIFY is one of multiple studies employing a variety of study designs and outcomes that will be necessary to fully understand if and what clinical consequences exist for those on ETI who may discontinue certain chronic maintenance therapies. The short duration of SIMPLIFY limits assessment of treatment withdrawal on long-term safety and clinical outcomes that can more easily be evaluated through patient-registry studies (29), including lung function decline and pulmonary exacerbations. Planning is currently underway in the United Kingdom for a randomized, registry-based, open-label study comparing 12-month changes in respiratory function for people with CF on established ETI therapy either continuing or reducing their treatment burden by removing hypertonic saline and/or dornase alfa from their daily care (CF STORM, EudraCT [European Union Drug Regulating Authorities Clinical Trials Database] number 2020-005864-77). The ability to follow long-term treatment patterns and clinical outcomes of SIMPLIFY participants through the CF Patient Registry will also enable important ancillary studies to investigate whether the decision to permanently stop treatment is related to disease course and compare outcomes across cohorts with varying treatment patterns. Importantly, the annual pulmonary-exacerbation rate among the CF population on ETI is now projected to be approximately 0.30 on the basis of long-term follow up of phase 3 trial participants, which represents a >60% reduction in previously recorded rates (30). This new “baseline” draws into question the role and clinical relevance of pulmonary exacerbation as a trial endpoint in future CF clinical trials enrolling individuals on ETI before they have developed advanced pulmonary disease (31). It would require 2,000 or more individuals to enroll and adhere to the assigned treatment regimen for at least a year for the trial to be adequately powered to detect modest differences in the risk of an acute pulmonary exacerbation between treatment groups. This is impractical in the setting of a randomized controlled trial in CF. Another limitation of treatment-withdrawal studies in general is that they are predicated on the assumption that there will be adequate adherence to ETI treatment. Although electronic monitoring of ETI adherence would be ideal, no currently available devices can monitor blister-pack medications. Daily ecological momentary assessment of ETI use was selected as a valid, self-reported approach for assessing adherence that minimizes recall bias, and the use of a run-in period will minimize the occurrence of significant nonadherence during SIMPLIFY. Lastly, although there is risk of selection bias toward healthier individuals being better candidates for treatment withdrawal, it is hoped that this bias will be mitigated among a homogeneous study population receiving established ETI therapy.

The SIMPLIFY design enables efficient use of the same trial infrastructure to address two questions under one protocol, recognizing that the window of opportunity to formally conduct a randomized trial may begin to close as individuals with CF on ETI experiencing clinical benefit may voluntarily withdraw these medications or alter their treatment frequency from daily to only as needed. It is expected that nearly 60–70% of the trial population will enter the trial on both hypertonic saline and dornase alfa on the basis of recent estimates from the CF Patient Registry, and the potential for these individuals to sequentially participate in both studies is likely to significantly decrease the overall burden on recruitment. The identical trial designs also enable pooling of data across studies to address several ancillary and important questions related to individuals on both therapies. In the planning phase for the study, alternative study designs were considered, including a crossover design that would markedly decrease sample-size requirements. The most significant challenge to this design, however, was the need for a short “wash-in” between study periods and the inability to confirm that the wash-in period was long enough to avoid carry-over effects from withdrawing in the first period. In addition, for studies in which safety outcomes are a key focus, it is very difficult to establish the time period to which adverse events (e.g., pulmonary exacerbations) should be attributed, particularly if they are delayed.

SIMPLIFY represents a major shift in CF clinical research from focusing on the additive effects of new therapies to improve clinical outcomes to now determining whether no meaningful changes will occur in these outcomes with the discontinuation of such therapies. Despite this distinction, trials testing new drugs and withdrawal studies like ours are both focused on trying to improve the lives of those with CF, and assuredly new and effective drugs will be needed going forward. Community engagement remains a necessity for determining the future of withdrawal trials in CF, including providing input on the therapies studied and key study-design attributes that promote ethical and feasible trial designs. This engagement must include people with CF (and their families) and medical teams who help to direct and monitor clinical care decisions, as so greatly benefited the development of this study. As a collaborative study linked to SIMPLIFY, the QUEST (Qualitative Understanding of Experiences with the SIMPLIFY Trial) study launched by the CFF Success with Therapies Research Consortium is enrolling subjects completing SIMPLIFY to characterize the perspectives of research participants about treatment withdrawal and treatment burden in the context of triple-combination CFTR-modulator therapy (NCT 04320381). The QUEST study is critical for providing patient-centered data to better understand the experience of those participating in withdrawal trials and factors that determine both participation and medication choices after the study. These data will also inform future medication- or therapy-withdrawal trials in CF. As is apparent in the design of SIMPLIFY, striking a balance between feasibility and ideal study-design principles is challenging yet can be achieved through prioritization of study aims and acknowledgment of limitations that will be necessary to account for in the interpretation of study results. With the broad input of the CF community, SIMPLIFY represents the largest multicenter, randomized, controlled medication-withdrawal study in the modulator era of CF. Careful interpretation of results from SIMPLIFY will provide timely evidence to inform important care decisions as to whether or not the daily treatment burden among those on CFTR-modulator therapy can now be considered. 
